# Wheat Chloroplast Targeted sHSP26 Promoter Confers Heat and Abiotic Stress Inducible Expression in Transgenic *Arabidopsis* Plants

**DOI:** 10.1371/journal.pone.0054418

**Published:** 2013-01-18

**Authors:** Neetika Khurana, Harsh Chauhan, Paramjit Khurana

**Affiliations:** Department of Plant Molecular Biology, University of Delhi, South Campus, New Delhi, India; National Taiwan University, Taiwan

## Abstract

The small heat shock proteins (sHSPs) have been found to play a critical role in physiological stress conditions in protecting proteins from irreversible aggregation. To characterize the hloroplast targeted *sHSP26* promoter in detail, deletion analysis of the promoter is carried out and analysed *via* transgenics in *Arabidopsis*. In the present study, complete assessment of the importance of CCAAT-box elements along with Heat shock elements (HSEs) in the promoter of sHSP26 was performed. Moreover, the importance of 5′ untranslated region (UTR) has also been established in the promoter *via Arabidopsis* transgenics. An intense GUS expression was observed after heat stress in the transgenics harbouring a full-length promoter, confirming the heat-stress inducibility of the promoter. Transgenic plants without UTR showed reduced GUS expression when compared to transgenic plants with UTR as was confirmed at the RNA and protein levels by qRT-PCR and GUS histochemical assays, thus suggesting the possible involvement of some regulatory elements present in the UTR in heat-stress inducibility of the promoter. Promoter activity was also checked under different abiotic stresses and revealed differential expression in different deletion constructs. Promoter analysis based on histochemical assay, real-time qPCR and fluorimetric analysis revealed that HSEs alone could not transcribe GUS gene significantly in *sHSP26* promoter and CCAAT box elements contribute synergistically to the transcription. Our results also provide insight into the importance of 5`UTR of *sHsp26* promoter thus emphasizing the probable role of imperfect CCAAT-box element or some novel *cis*-element with respect to heat stress.

## Introduction

High temperature stress is one of the most common abiotic stress among many of the world crops, reducing both yield and quality of crops, and there is a need to increase productivity for warmer areas of the world. Worldwide several breeding and molecular approaches are being utilized to impart heat tolerance in crop cultivars. It is known that plants synthesize a set of evolutionary conserved proteins called Heat Shock Proteins (HSPs) upon heat stress, and many groups have produced thermo tolerant plants by overexpressing these HSPs. The HSP family has been classified into five groups depending on their molecular weight: HSP100, HSP90, HSP70, HSP60 and small HSPs [1]. The expression level of *Arabidopsis thaliana* Heat Shock Factor (AtHSF) was successfully altered and thus HSPs were overexpressed in *Arabidopsis* plants [2]. These small HSPs are known as stress proteins and these stress proteins were found to protect photosynthesis in cells during various abiotic stresses like heat, salt, drought, osmotic, oxidative, and other photoinhibitory stresses [3]–[11]. Small HSPs has also been found to be regulated at specific plant developmental stages like embryogenesis, fruit maturation and pollen development, other than abiotic stresses [12]–[13]. Jiang et al. [14] characterized *RcHSP17.8,* a cytosolic class I sHSP, from *Rosa chinensis* by producing transgenic *Arabidopsis thaliana* plants that constitutively expressed RcHSP17.8 and these plants exhibit increased tolerance to multiple abiotic stresses such as heat, salt, osmotic and drought stress. They also showed the same effect in *Escherichia coli* and yeast by overexpressing recombinant RcHSP17.8 in both these species as well.

A powerful and more sensitive approach for measuring the activity of any heat shock promoter is by fusing the promoter of a plant heat shock gene to GUS (β-glucuronidase) reporter gene thereby allowing to measure the developmental and tissue specific expression with and without heat stress [15]–[16]. Transgenic *Arabidopsis* plants were produced by Takahashi et al. [17] which contained the promoter of HSP18.2 gene fused to the GUS gene and histochemical analysis was carried out. They showed that heat stress induced the GUS gene activity in almost all the organs of the plant. Similarly, heat shock induced GUS activity was also observed in transgenic *Arabidopsis* plants when the promoter of HSP 81–1 gene was fused to the GUS gene [18]. Crone et al. [19] did a detailed analysis of the expression of the GUS gene when fused with small heat shock protein gene promoter, *Glycine max HSP17.5E* (*GmHSP17.5E*) in all the organs and tissues of the flower as a function of development with and without heat stress. They found that the promoter of *GmHSP17.5E* is not uniformly expressed after heat shock in different floral tissues. For example, expression could be seen in all the developmental stages of sepals but not in petals, and the expression is even complex in pistil and anthers. They observed GUS expression in style and upper portion of ovary, but not in lower part of ovary or ovules. Similarly in stamens, GUS induction could be seen only in filament or in the vascular tissue from the filament into the anther but not in other tissues of anther or microspores. However, in vegetative tissue, heat shock induces its response in all the tissues and organs of young plant. A detailed study to examine GUS activity in different organs at different temperatures was done by Moriwaki et al. (16). They observed the expression pattern of the Arabidopsis HSP 18.2-GUS gene chimera at the recovery period following heat shock treatment in transgenic *Nicotiana plumbaginifolia*. They optimised the HS temperature in anthers, petals and capsules to be 42°C; in immature seeds, it is 36°C; in placentas of capsules, it is 39°C. Thus, they showed organ and different developmental stages specific heat stress inducibility. The usefulness of a heat shock promoter for studying gene functions and also for studying *cis*-acting transcriptional elements is discussed in detail in transgenic zebrafish [20], where HSP70 promoter has been used for manipulating transgenes in zebra fish embryos.

Rice MT (Metallothionein) promoter has been analyzed in transgenic *Arabidopsis* using GUS as a reporter [21]. Six promoters of seed storage glutelin gene showed the expected spatial expression pattern within the endosperm [22]. Full length or truncated pine ACC oxidase gene promoters showed distinct patterns of expression when responded to IAA (Indole-3-Acetic Acid) and wounding stress [23]. The promoter of *Arabidopsis thaliana* gene *AtGILTpro*- (Gamma Interferon-responsive Lysosomal Thiol reductase) was fused to the *uid*A reporter gene and was selected as a useful seed coat outer integument (including mucilaginous layer)-specific promoter for canola [24]. The histochemical advantage of GUS fusion to *Arabidopsis* CORI3 (CORONATINE INDUCED) promoter also revealed two integrated *cis*-regulatory regions required for transcriptional activity in companion cells [25]. Similarly, full length promoter fragments from lemon and lime were investigated by fusing them to GUS reporter gene followed by their transient transformation in tomato floral organs [26]. Promoter analysis of Chalcone synthase from *Populus trichocarpa* showed that it is capable of directing GUS gene expression in both wounded and unwounded leaves [27]. Three different promoters could also induce GUS expression in abiotic stress like ABA and salt treatments in both vegetative and floral organs in transgenic rice [28].

HSP26 has been well characterized from *Saccharomyces cerevisiae* [29]. Chaperone assays were performed at different temperatures that show that there is temperature dependent dissociation of HSP26 complex into smaller active species and then reassociation of this complex for functional activation of this chaperone [30]. The thermodynamic and kinetic characteristics of structural changes when HSP26 is heat activated showed that its temperature sensing is a function of its middle domain that changes its confirmation in response to temperature [31]. To determine the role of chloroplast localised small HSP26 in heat sensitive and heat tolerant variant of bentgrass (*Agrostis stolonifera* cv. *palustris*), different isoforms of HSP26 gene were isolated and their structure and expression were characterized [32].

In a previous study from our lab, we have cloned chloroplast targeted *sHSP26* from bread wheat (*Triticum aestivum)* and characterized it *via* transgenic *Arabidopsis* plants [33]. Transgenic *Arabidopsis* plants overexpressing *sHSP26* were shown to be substantially tolerant than wild type plants under continuous moderate high temperature regimen. The HSP26 promoter was also functionally characterized in rice transgenics. In the present study, the promoter of *TaHSP26* is characterized by deletion analysis of the promoter *via Arabidopsis* transgenics confirming the inducibility of this promoter under heat and other abiotic stresses.

## Results

### Sequence Analysis of the *TaHSP26* Promoter

A 1514 bp *TaHSP26* promoter with 112 bp 5′ UTR is designated as 1625 bp full length promoter that was reported in the previous study by Chauhan et al. [33] by PLACE database [34] (http://www.dna.affrc.go.jp/PLACE) (Fig. S1). Promoter analysis revealed the presence of several transcription factor-binding sites associated with various environmental signals. For example- there are several MYC-rd22 sites in the promoter that respond to drought stress and ABA (abscisic acid) signalling. The upstream region of *TaHSP26* contains all the three types of HSEs; Perfect-type (nGAAnnTTCnnGAAn), Gap-type (nGAAnnTTCn(5 bp)nGAAn and Step-type (nTTCn(5 bp)nGAAn(5 bp)nTTCn) [35]–[37]. Two Stress Responsive *cis*- acting Elements (STREs) (AGGGG) were found at −599 bp and −781 bp, believed to be involved in mediating the general stress response. These sequences are found in the promoter of many stress-responsive genes and are found to be induced under heat stress, osmotic stress, low pH and nutrient starvation [38]. Three CCAAT- box elements were also found in this promoter at −721 bp, −1209 bp and −1435 bp sites, respectively. In an earlier report, these CCAAT box regions were reported to be essential for gene expression, while deletion of the CCAAT box region (deletion −81/−63) reduced the strength of the *nos* promoter by many folds [39].

### *TaHSP26* Promoter Deletion Constructs

To gain further insights into the functional role of *TaHSP26* promoter region, a series of deletions were made by designing primers that truncate promoter fragments. Since CCAAT box elements were found to enhance expression of chimeric heat shock genes in transgenic tobacco [40], a series of deletions were generated removing CCAAT box-elements gradually. Moreover, to assess the importance of 5′ Untranslated Region (UTR), UTR region was completely deleted in some of the constructs. Thus, a total of eight constructs were generated; [Full Promoter with UTR (Pro26+UTR), full promoter without UTR (Pro26–UTR), Del 1, Del 2, Del 3, Del 4, Del 5 and Del 6].

- **Full Promoter with UTR** - 1625 bp - includes all the known stress and development related elements, i.e. 3 CCAAT box, 3 HSEs, 2 STREs, 5 Myc- rd22 and UTR.
- **Full Promoter without UTR** - 1514 bp - includes all the known stress and developmental related elements, 3 CCAAT box, 3 HSEs, 2 STREs, 5 Myc- rd22 but UTR deleted.
- **Del 1** - 1302 bp - includes 2 CCAAT box, 3 HSEs, 2 STREs, 5 Myc- rd22, 1 CCAAT box deleted and includes UTR.
- **Del 2** - 885 bp - includes 1 CCAAT box, 3 HSEs, 2 STREs, 2 Myc- rd22, 2 CCAAT box deleted and includes UTR.
- **Del 3** - 530 bp - includes no CCAAT box, 3 HSEs, no STREs, no Myc- rd22, all 3 CCAAT box deleted and includes UTR.
- **Del 4** - 1190 bp - includes 2 CCAAT box, 3 HSEs, 2 STREs, 5 Myc- rd22, 1 CCAAT box and UTR deleted.
- **Del 5** - 773 bp - includes 1 CCAAT box, 3 HSEs, 2 STREs, 2 Myc- rd22, 2 CCAAT box and UTR deleted.
- **Del 6** - 418 bp - includes no CCAAT box, 3 HSEs, no STREs, no Myc- rd22, all 3 CCAAT box and UTR deleted.

**Figure 1:**
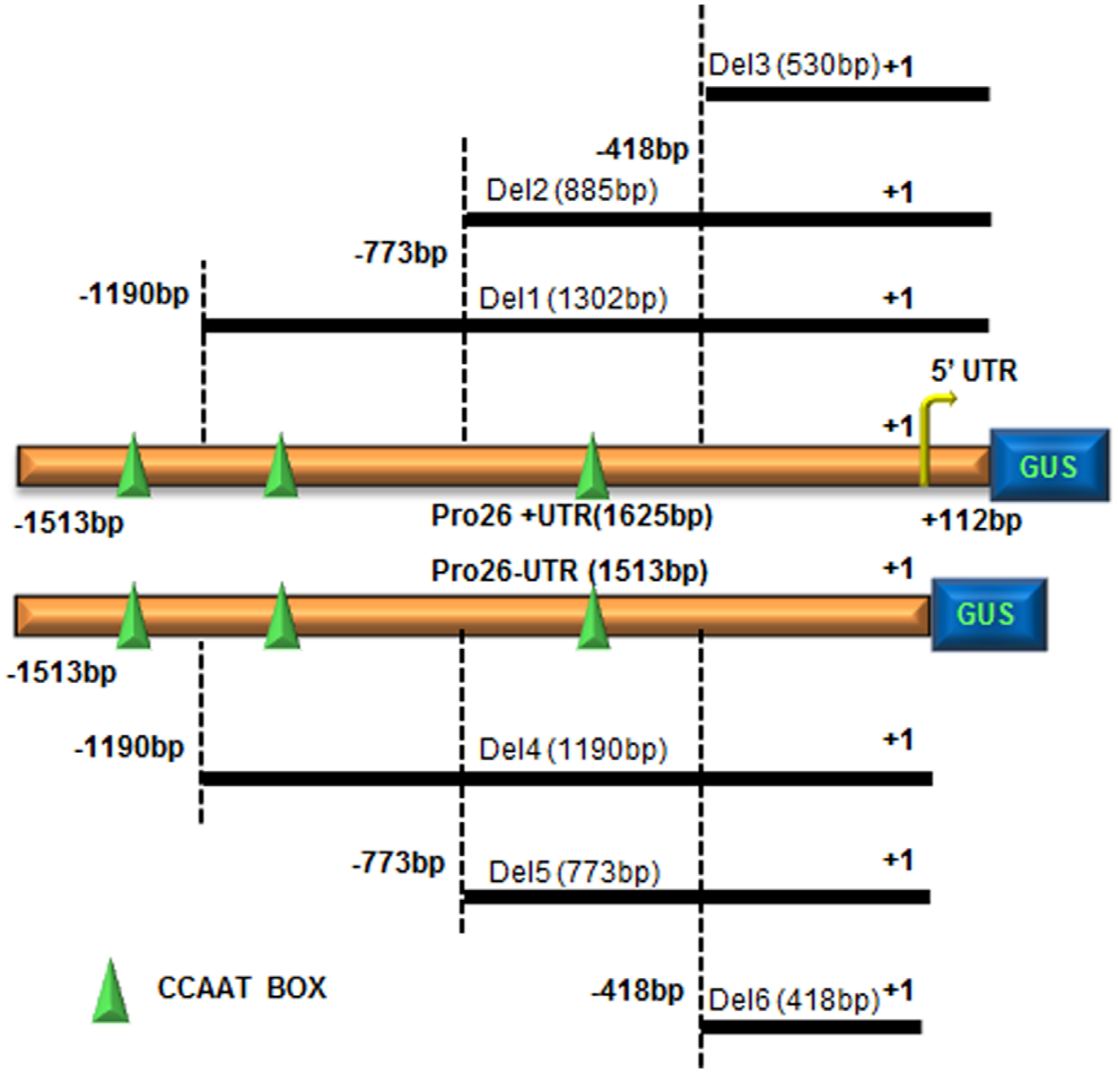
Schematic representation of different *TaHSP26* promoter deletion constructs used for transformation of *Arabidopsis* plants. The numbers at the 5′end indicate the lengths of the upstream end of the different promoter deletion constructs, the downstream end consist of 112 bp UTR in four of the constructs. The green symbol represents the CCAAT BOX1 *cis*-acting element present in the promoter.

Schematic representations of all the *TaHSP26* deletion constructs used for transformation of *Arabidopsis* are shown in Fig. 1. All these constructs were PCR amplified using region-specific primers from *TaHSP26* promoter (Fig. S2) and cloned into plant transformation GATEWAY ™ vector pMDC164 mediated by pENTR™/D-TOPO. The constructs were then transformed in *Arabidopsis via* floral dip method [41].

### *TaHSP26* Promoter Activity in Plants after Heat Stress

The temporal and spatial distribution of GUS in *TaHSP26* promoter carrying *Arabidopsis* plants were investigated in T_4_ generation grown in culture-room conditions. Transgenic plants harbouring full-length promoter of *TaHSP26* gene were analysed histochemically under control and heat-stressed conditions (37°C, 2 hrs). Full seedlings were observed for GUS expression; a blue colored end product was observed exclusively in the heat-stressed transgenics and no GUS activity was detected in seedlings of control plants. Shoot and root tissues of full-length promoter transgenics showed intense GUS staining, sections of both the tissues were observed under bright field using fluorescence microscope (Leica, Germany) (Fig. S3). Three independent transgenic lines with consistently high levels of GUS expression were selected for further analysis.

### Effect of Different Deletions of *TaHSP26* Promoter on Heat-shock Responsiveness in Transgenic *Arabidopsis*

**Figure 2:**
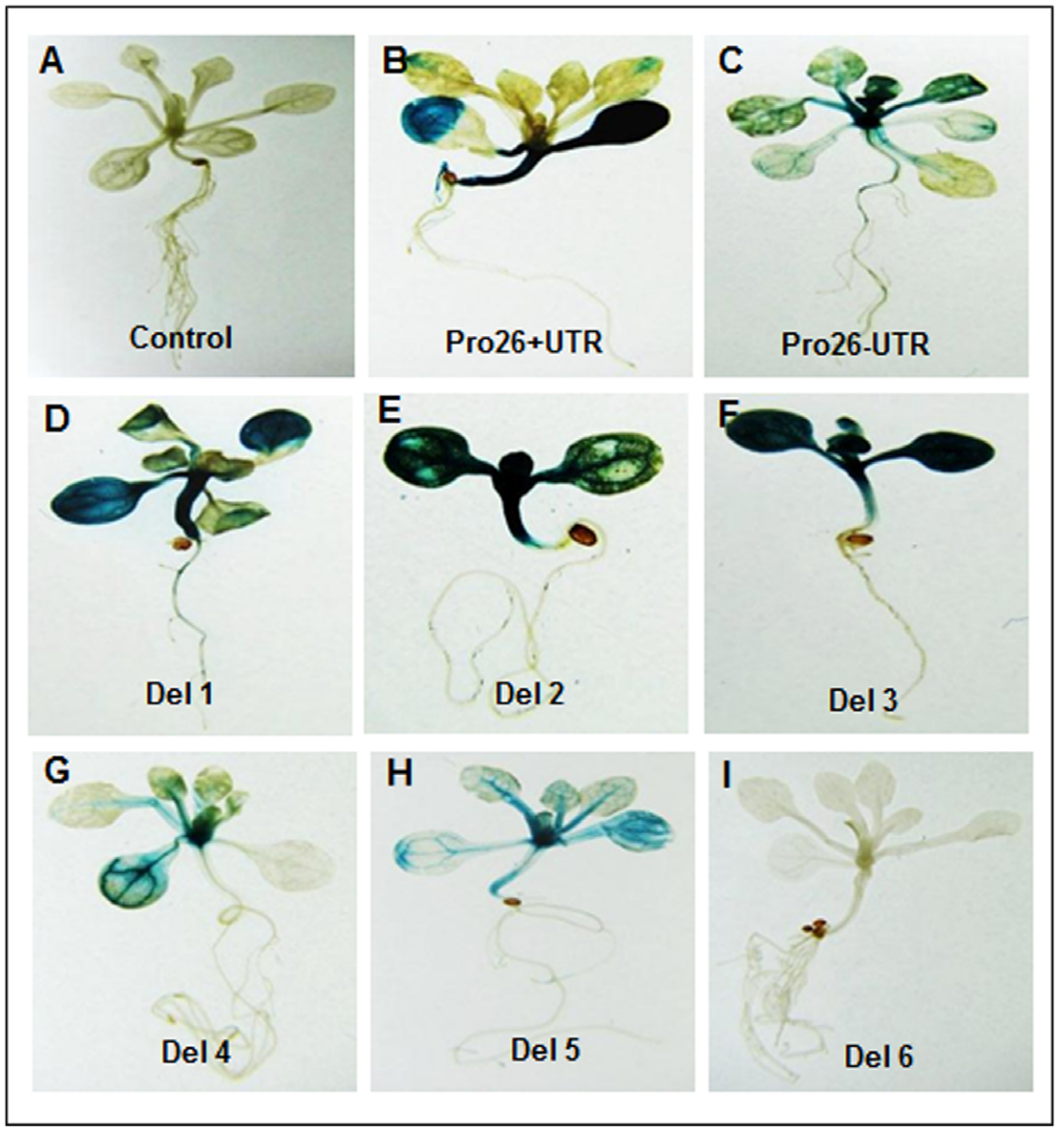
Histochemical localization of GUS gene activity in transgenic *Arabidopsis* plants containing full *TaHSP26* promoter with and without 5′ UTR and six different deletion constructs in two-week-old seedlings. (a) Control (transgenic without heat stress); (b–i) transgenics with heat stress at 37°C for 2 hrs; (b) *TaHSP26* promoter with UTR; (c) *TaHSP26* promoter without UTR; (d–f) Del 1, 2, 3 with UTR; (g–i) Del 4, 5, 6 without UTR.

In continuation to the Pro26+UTR and Pro26-UTR constructs, 6 deletion constructs were also undertaken to measure the TaHSP26 activity under various abiotic stresses. Deletion constructs were analysed histochemically in three-independent transgenic lines for each construct. The results showed that plants harbouring construct Del-1, 2 and 3 showed a remarkable increase in the GUS expression in comparison to Del-4, 5 and 6, but, the GUS levels of all these constructs were relatively low when compared to the full-length promoter (Fig. 2d–f). Among Del-1, 2 and 3, Del-1 showed higher amount of GUS activity in terms of intense blue colour followed by Del-2 and then, Del-3. Histochemical analysis showed that further deletions of the promoter (in absence of UTR) results in gradual reduction of GUS gene expression relative to full-length promoter without UTR, Pro26-UTR (Fig. 2g & h). Among Del-4 and Del-5, Del-4 showed comparatively higher GUS levels compared to Del-5. Further reduction of the promoter size to 418 bp, resulted in complete absence of GUS activity in Del-6 (Fig. 2i). Thus, plants carrying progressive deletions of the promoter resulted in decreasing pattern of GUS activity and ultimately no detectable GUS. The control plants did not show any GUS activity and the three lines of respective deletion constructs analysed by GUS histochemical assay showed similar expression pattern.

### GUS Transcript Activity by *TaHSP26* Promoter and its Deletions in Response to Heat Stress

**Figure 3:**
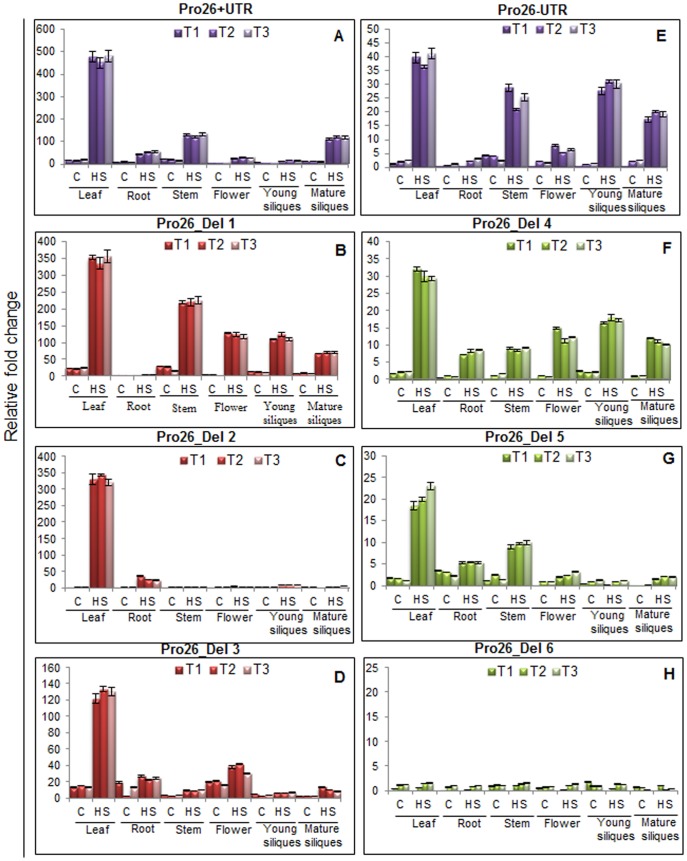
Analysis of Pro26 promoter activity in different tissues of two-week-old transgenic *Arabidopsis* plants. GUS transcript was analyzed by quantitative RT-PCR in full promoter as well as all the deletions lines. Three individual transgenic plants (T1, T2, T3) from each line were analyzed. Standard error bars are shown.

To analyse the GUS at the transcriptional level, quantitative RT-PCR was performed in two-week-old transgenic *Arabidopsis* plants. Various organs analysed were leaf, root, stem, flower, young silique, mature silique in control as well as HS (37°C, 2 hr) plants to determine organ specificity (if any) of *TaHSP26* promoter deletion constructs (Fig. 3) in three independent transgenic lines of each construct. Compared to the control conditions, a drastic increase in gene expression was observed when heat-shock was given to *Arabidopsis* transgenic plants. In Pro26+UTR construct, where highest GUS activity was observed, GUS transcript was found to be up-regulated about 450- folds in stressed leaf tissue as compared to the control leaf tissue (Fig. 3a). GUS transcript also showed highest upregulation in heat-stressed stem and mature silique tissue. GUS transcript was also more visible in heat-stressed root and flower tissue. This full promoter of *TaHSP26* with UTR contained many stress and developmental related elements, i.e. 3 CCAAT box, 3 HSEs, 2 STREs, 5 Myc- rd22. The high GUS induction response could be due to a synergistic effect of heat-stress responsive elements in the promoter. In construct Del-1 of *TaHSP26* promoter, it was observed that the GUS transcript has reduced by the deletion of one CCAAT box present at position −1435. There is a 100-fold reduction of GUS transcript in Del-1, when compared to the full-length promoter (Fig. 3b). The leaf tissue upon heat-stress showed GUS transcription induction about 350-fold higher as compared to the control leaf tissue. GUS transcript was also many folds higher in heat-stressed stem, flower, young siliques, and mature siliques. Further deletion of the promoter to point −885, results in similar decrease in GUS levels. In Del-2, GUS expression levels also showed up-regulation by 300-fold when compared to control in leaf tissue, but there is a decrease in GUS gene expression by 50 fold as compared to Del-1 construct (Fig. 3c). The Del-2 promoter region contained 1 CCAAT box, 3 HSEs, 2 STREs, 2 Myc- rd22 and UTR. The deleted promoter part contained 2 CCAAT boxes, at position −1435 and −1209. In Del-3 construct, GUS transcript levels further reduce to many folds when the third CCAAT box present in the promoter, at −721 position, also got deleted (Fig. 3d). Del-3 promoter region included only HSEs specifically while other stress-responsive elements like STREs, Myc- rd22 were deleted along with CCAAT box. However, 5′ UTR was present in all the constructs discussed so far.

In Pro26-UTR construct, where UTR was deleted in the full-length promoter, GUS expression showed a drastic reduction as compared to the full length promoter with UTR (P+UTR) as was also shown histochemically (Fig. 2c). The construct showed up-regulation of GUS transcript in response to HS by 40-folds in leaf tissue, however, it is about 350-folds lower when compared to Pro26+UTR construct (Fig. 3e). The construct included all the known stress and developmental related elements, 3 CCAAT box, 3 HSEs, 2 STREs, 5 Myc- rd22 but without UTR. It also showed significant levels of GUS transcript in heat-stressed stem, young siliques and mature siliques. Deletion of the fragment which contained 1 CCAAT box from Pro26-UTR formed Del-4 construct. In agreement with the earlier observation made by histochemical assay, deletion of the CCAAT box resulted in decrease in GUS transcript level by 10-folds, when compared to full Pro26-UTR construct (Fig. 3f). GUS transcript was also induced in rest of the tissues studied, i.e., root, stem, flower, young siliques and mature siliques. The fold change, however, was low in almost all the tissues. Gene expression levels decrease further when the promoter is deleted to point −773 thus excluding 2 CCAAT boxes, in addition to UTR. Del-5 thus consisted of 1 CCAAT box, 3 HSEs, 2 STREs and 2 Myc- rd22. GUS transcript level goes further down by 10-fold when compared to Del-4 construct as is evident in heat-stressed leaf tissue (Fig. 3g). In Del-6, deleting the promoter fragment to −418 bp resulted in abolishing CCAAT box, STREs and Myc- rd22. Only the HSEs were present in this shortest promoter deletion that also excludes UTR. Interestingly, it was observed that GUS transcription reduced to the marginal levels if any, almost indistinguishable to that of control (Fig. 3h). Thus, we infer that without UTR and with no CAAT box element present in the shortest promoter fragment, HSEs alone could not transcribe GUS gene significantly.

### Quantitative Estimation of GUS Driven by *TaHSP26* Promoter and its Deletions in Response to Heat Stress

**Figure 4:**
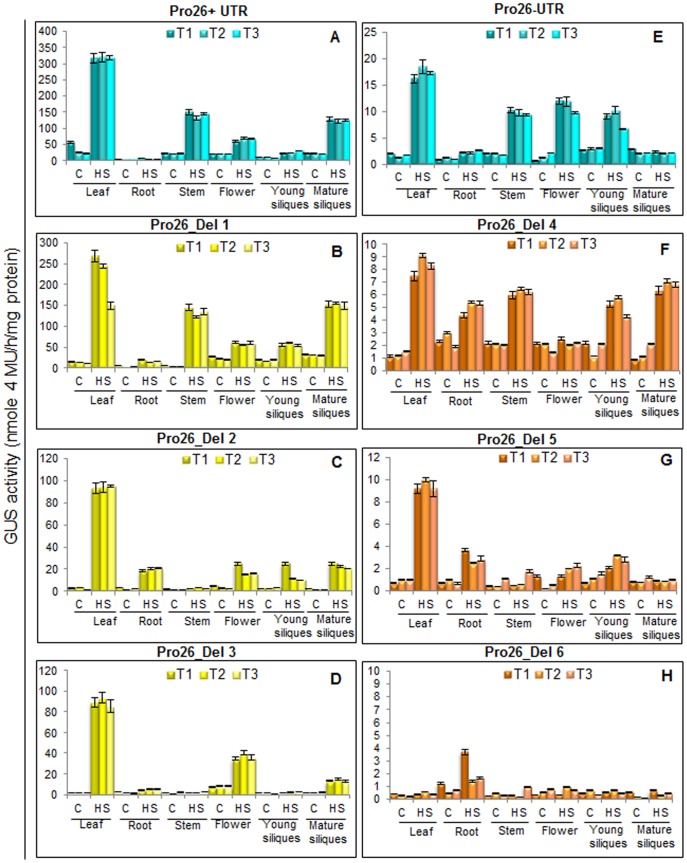
Analysis of Pro26 promoter activity in different tissues of two-week-old transgenic *Arabidopsis* plants. Quantitative measurement of GUS activity was determined using protein extracts from different tissues. Three individual transgenic plants (T1, T2, T3) from each line were analyzed. Standard error bars are shown.

Quantitative measurement of GUS activity was also determined in two-week-old *Arabidopsis* transgenics plants. The same tissues used for real-time PCR for GUS transcript were used for protein extraction, i.e. leaf, root, stem, flower, young silique and mature silique in transgenic control as well as heat stressed (37°C, 2 hr) plants (Fig. 4). Three independent transgenic lines for each construct were analysed for fluorimetric estimation of GUS protein. Three technical replicates were also taken for each tissue analysed. The activity of GUS was expressed in nmol of 4-MU/mg protein/h. The results quantitate the GUS protein and revealed that the highest amount of GUS activity of 300 units was observed in full-length promoter with UTR, Pro26+UTR (Fig. 4a). All the deletion fragments that contained UTR (Del-1, Del-2, and Del-3), progressive deletion of the three CCAAT box elements resulted in decrease of GUS protein as quantified by the fluorimetric values of 250, 100 and 80 units (Fig. 4b–d). Deletion of UTR from the full-length promoter resulted in a dramatic decrease in GUS protein levels; a decrease of 300 units as compared to the full-length promoter (Fig. 4e). Further, gradual deletion of the three CCAAT box elements from the promoter in absence of UTR (Del-4 and Del-5), resulted in even more reduction in the GUS protein level (Fig. 4f–g). In Del-6, though histochemical staining did not show any GUS expression, yet the fluorimetric analysis revealed some marginal GUS activity (Fig. 4h).

### 5′ UTR Mediated Expression of *TaHSP26*

To assess the possible involvement of 5′ UTR in the regulation of expression of *TaHSP26*, two expression cassettes of full-length promoter were generated; one with UTR and other without UTR. Ten-independent transgenic lines from each construct were analysed for the presence of GUS gene expression. Three lines with consistent expression for the GUS gene were selected for further analysis.

**Figure 5:**
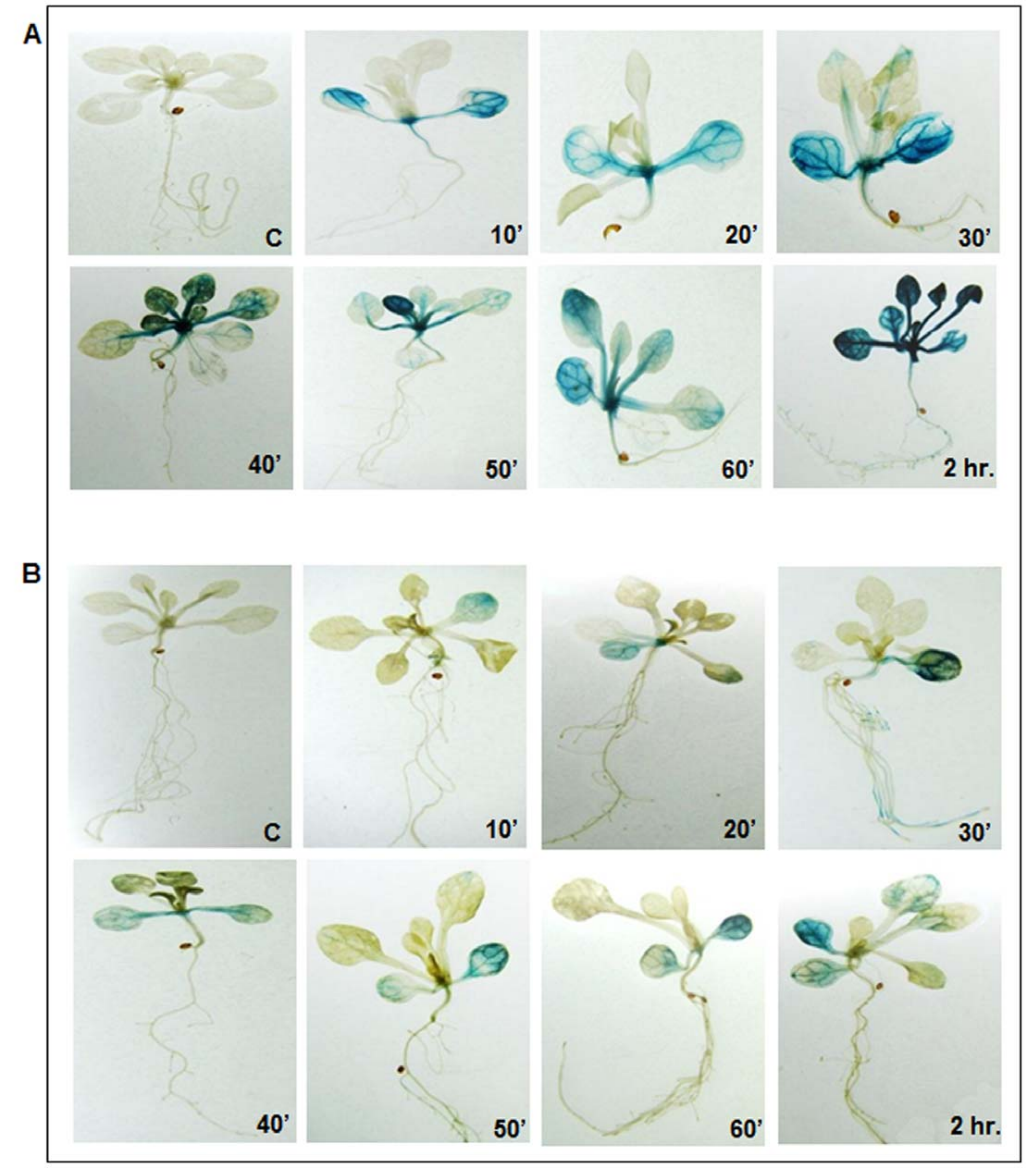
GUS histochemical assay showing induction of GUS gene governed by *TaHSP26* promoter in three-week-old *Arabidopsis* transgenics when heat stressed at different time-points. A. Pro26 promoter with UTR showed GUS activity at different time points of heat stress. B. Pro26 promoter without UTR showed GUS activity at different time points of heat stress. Control taken is transgenic *Arabidopsis* plant without heat stress. C = Control (non heat-stressed transgenic), HS = Heat Stress (37°C, 2 hrs.).

The levels of GUS activity were assayed histochemically in the Pro26+UTR and Pro26-UTR constructs in three-week-old *Arabidopsis* seedlings when heat-stressed at 37°C for different time durations. The results showed that transgenic plants harbouring *TaHSP26* promoter with or without UTR showed immediate appearance of the blue colour of GUS activity as early as 10 min; while no GUS staining was observed under control conditions (Fig. 5). However, a visibly reduced GUS staining was observed for transgenic plants harbouring full length promoter without 5′ UTR as compared to transgenic plants harbouring full-length promoter with 5′ UTR. Increase in the duration of heat stress at an interval of every 10 min till 2 hrs. was given and seedlings were observed histochemically. Consistent with the earlier observations, transgenic plants harbouring full-length promoter with UTR showed progressive increase in the GUS activity and the plants showed maximum GUS activity after 2 hrs of HS (Fig. 5A). Similarly, plants harbouring full length promoter without UTR also showed an increase in the GUS expression and the induction was maximum again at 2 hrs (Fig. 5B). However, there was a visible difference in the intensity of blue colour in both the constructs at all the different time points of heat stress.

**Figure 6:**
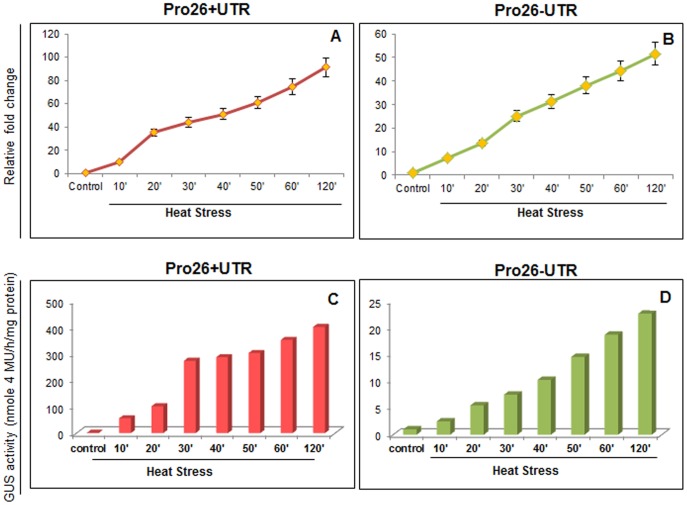
Analysis of promoter activity in three-week-old *Arabidopsis* transgenic plants after heat stress was given at different time intervals. GUS transcript was analyzed by quantitative RT-PCR and GUS protein was analyzed by fluorimetry in (a & c) Full promoter with UTR; (b &d) Full promoter without UTR.

Quantitative RT-PCR of *GUS* transcript was also analysed of both the constructs (Pro26+UTR, Pro26–UTR) for three-week-old *Arabidopsis* seedlings that were frozen immediately at −80°C after heat stress was given to them at various intervals. The analysis revealed that under the same heat-stress durations, construct Pro26+UTR showed a 20-fold-increase in expression when compared with the construct Pro26-UTR after 2 hrs of HS (Fig. 6a–b). Quantitative estimation of the GUS protein revealed that deletion of the UTR resulted in reduced induction of the GUS reporter gene by approx. 50-fold (Fig. 6c–d). After 2 hrs of HS, Pro26+UTR construct showed GUS activity of 400 nmole 4 MU/hr/mg protein, while that of Pro26-UTR construct showed GUS activity of 22.6 nmole 4 MU/hr/mg protein. Thus, a dramatic difference in gene-expression was observed when UTR was deleted from *TaHSP26* full-length promoter.

### Activity of *TaHSP26* Promoter and its Deletions under other Abiotic Stresses

**Figure 7:**
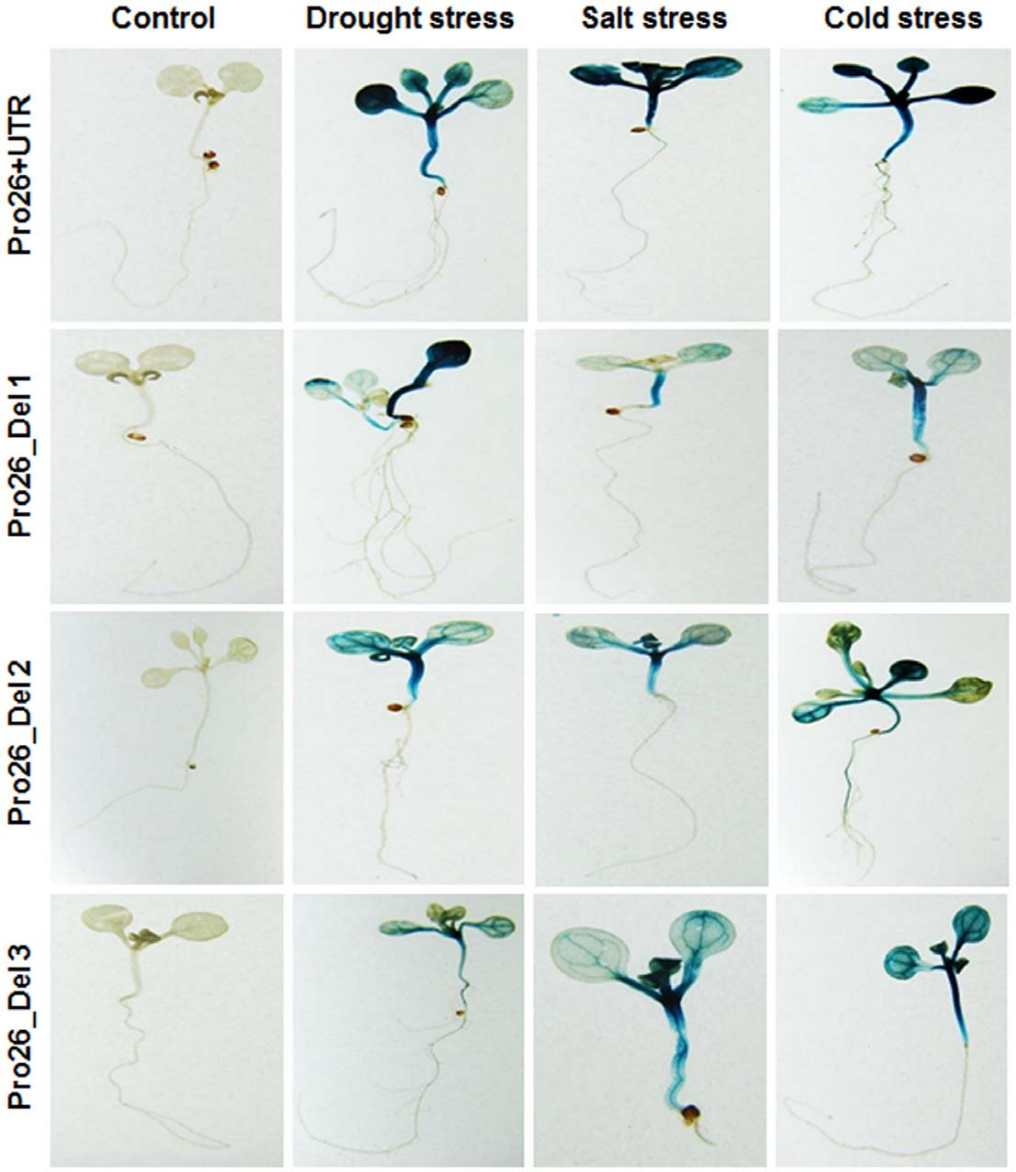
Analysis of Pro26 promoter activity in two-week-old *Arabidopsis* transgenic seedlings under three different abiotic stresses. Transgenic lines with UTR were analyzed for simulated drought (mannitol 2%, 24 hr), salt (150 mM, 24 hr) and cold stress (4°C 24 hr).

To carry out a comparative analysis of *TaHSP26* promoter and its deletion constructs under different abiotic stresses, GUS reporter gene activity was investigated histochemically in two-week-old *Arabidopsis* transgenic seedlings. Plants were treated with three different abiotic stresses for 24 hrs: for drought stress, plants were exposed to 2% mannitol; for salt stress, plants were treated with 150 mM salt (NaCl) solution; for cold stress, plants were kept at cold room (4°C) for 24 hr. Next day, plants were incubated overnight in GUS assay buffer at 37°C and histochemically analysed. Control plants were not treated to any of the abiotic stresses, and did not show GUS staining in any of the promoter constructs. Histochemical analysis showed that the constructs with UTR responded tremendously to drought, salt and cold stress as well. The blue colour of GUS activity was highest in the full-length promoter (Pro26+UTR), and to lesser levels in other deletion constructs also (Fig. 7).

**Figure 8:**
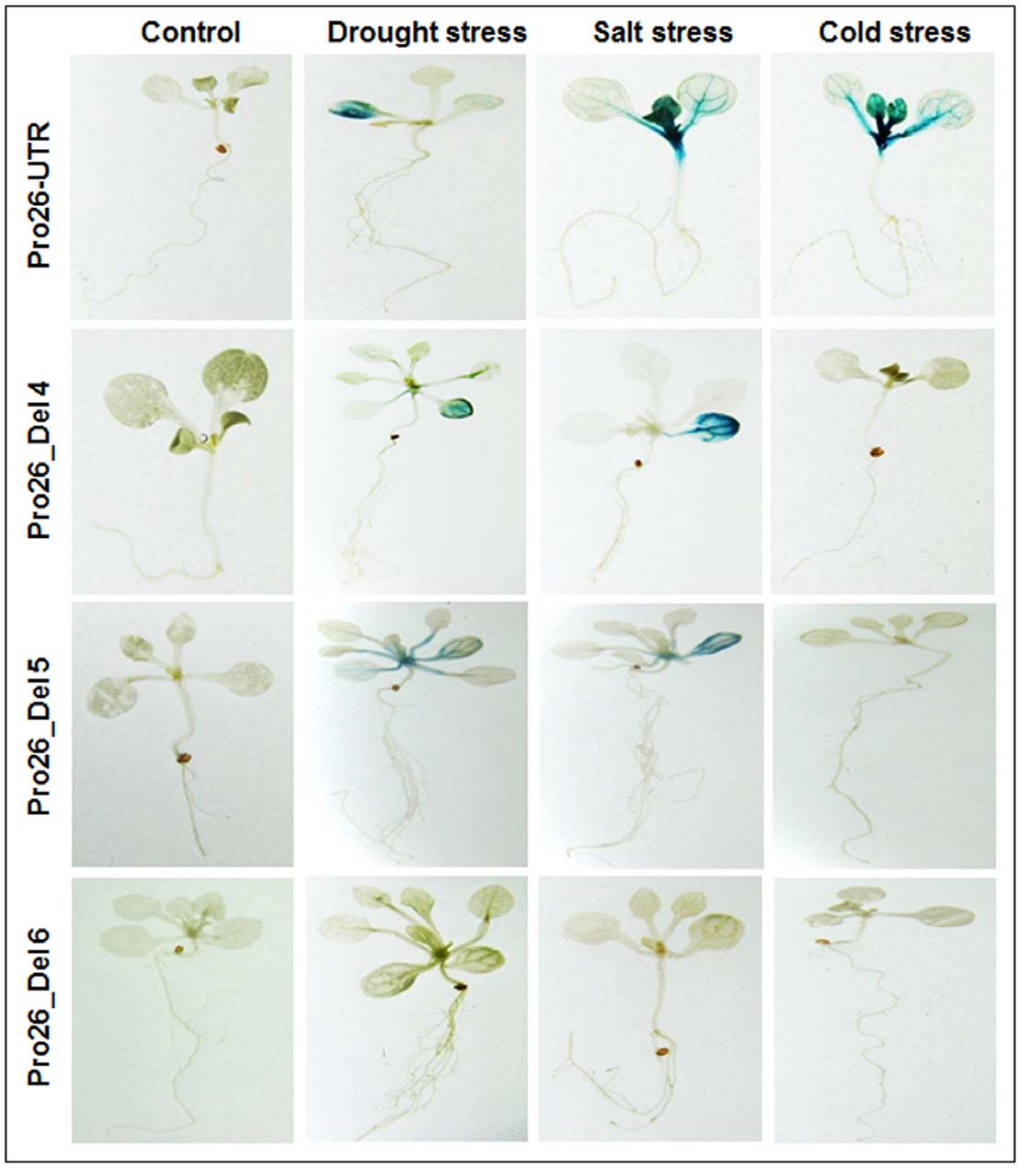
Analysis of Pro26 promoter activity in two-week-old *Arabidopsis* transgenic seedlings in three different abiotic stresses. Transgenic lines without UTR were analyzed for simulated drought (mannitol 2%, 24 hr), salt stress (150 mM, 24 hr) and cold stress (4°C, 24 hr).

GUS activity was also checked in full-length promoter construct without UTR and the promoter deletions that are without UTR (Del-4, Del-5 and Del-6) (Fig. 8). It was observed that deletion of UTR from the promoter caused a decrease in the GUS staining in all the three abiotic stresses (drought, salt, and cold). This again reveals the importance of UTR or other *cis*-element present in UTR that are responsive to abiotic stresses. Construct Del-4 and Del-5 showed GUS induction in drought stress and salt stress but no GUS staining was observed in cold stress. However, in Del-6 construct, no GUS levels were detected in any of the three stresses studied. In all *TaHSP26* promoter constructs, histochemical staining of two-week-old *Arabidopsis* seedling under simulated drought and salt stress displayed a similar GUS staining pattern as was in the case of heat-shock treated plants. However, only in case of cold stress, there was differential expression observed in case of Del-4 and Del-5 and no GUS induction was observed.

## Discussion

We have previously analysed the expression of a wheat chloroplast targeted sHSP, *TaHSP26* in different tissues of wheat representing major growth stages and abiotic stresses [42]. Along with vegetative tissues, *TaHSP26* transcript was found to be highly inducible by heat-stress in flower and developing seed tissues. Role of *TaHSP26*, in conferring heat stress tolerance and during seed development has been shown earlier by Chauhan et al. 2012 [33]. In the present study, promoter of *sHSP26* is further characterized by deletion analysis *via Arabidopsis* transgenics and confirming the inducibility of the deletion constructs in heat stress. Not many reports are available that allowed gene expression in transgenic plants induced only by external factors. One such report by Freeman et al. [43] used GUS reporter gene to demonstrate the heat induction of barley *Hvhsp17* gene promoter in transgenic wheat. Gus gene was induced only in heat stressed tissue and was expressed in all tissues and organs tested.

Based on our results as shown by histochemical assay, quantitative RT-PCR and fluorimetric analysis, it could be inferred that without UTR and with no CAAT box element present in the shortest promoter fragment, HSEs alone could not transcribe GUS gene. As was also shown by Haralampidis et al. [44] the *cis*-elements present in promoter region of *AtHSP90-1* contribute in a combinatorial manner to regulate the expression in development, suppression, or stress conditions. They concluded that the two stress responses (heat stress and arsenite treatment) may involve common but not necessarily the same regulatory elements. Our results clearly demonstrate that *TaHSP26* promoter is highly heat inducible and Heat Shock Elements (HSEs) alone are not sufficient for heat-shock induciblity, CCAAT box elements contribute synergistically to the transcription of heat shock genes. Same was reported earlier by Rieping and Schoffl (1992) [40] in soybean *Gmhsp 17.3* promoter. Heat inducible CAT (chloremphenicol acetyltransferase) activity was detected when three CCAAT boxes and a single HSE were reconstituted in the HS promoter; however deletion of the CCAAT box sequences reduced CAT activity five-folds. Deletion of the CCAAT box region reduced the *nos* promoter strength many folds as was shown earlier by An (1987) in transgenic tobacco [39]. The CCAAT box elements are one of the most common regulatory elements present in 30% of eukaryotic promoters and are conserved in promoters of the heat-shock genes [45]. This fact was thoroughly studied in promoters of HSP70, which are the most well studied among heat shock genes [46]. In yeasts, plants and mammals, NF-Y binds to CCAAT box in most of the promoters and activates it [47]. The importance of CCAAT box has been shown by Landsberger et al 1995 where they conclude that CCAAT box maintains the promoter in open chromatin configuration so that HSF could rapidly activate after thermal stress [48]. *In vivo* footprinting experiments in mouse cells by Abravaya et al. [49] showed that CCAAT box elements are constitutively protected prior to the heat shock whereas HSEs bound to the HSFs after heat shock has been given. In CCAAT-less constructs, the promoter remains in a closed nucleosomal configuration, thus does not allow HSFs to bind to HSEs and activate transcription after heat stress induction [48]. Mutation analysis has also showed that the basal transcriptional activity of human Hsp70 promoter *in vitro* was primarily dependent on the CCAAT-box element located at −65 [50]. Thus, as evident by our results, we can also hypothesize that in wheat plants, CCAAT box elements may contribute in maintaining the open chromatin configuration so as to allow HSFs to bind to HSEs after heat induction. Also, in the case of *TaHSP26* promoter, 5′ UTR has contributed significantly to the heat-shock inducibility. This indicates that the heat-stress responsive elements required for the expression of the gene are also located in this region which needs to be characterized further.

Similarly, importance of HSE for the heat shock induction of the *apx1* gene was confirmed by mutational analysis [51]. *In vitro* analysis of the interaction between tomato HSF and the *apx1* promoter confirmed that HSE represents a functional HSF-binding site. Thus, confirming that HSE is responsible for the heat shock induction of the gene and also contributing partially to oxidative stress induction. Also, developmental induction of *HaHsp17.6G1* promoter was abolished when any mutation was performed in its HSE [52].

Our results have also shown a dramatic difference in gene-expression when UTR was deleted from *TaHSP26* full-length promoter. In a similar report, Karthikeyan et al. [53] studied the effect of 5′ UTR intron and the role of putative *cis*-elements present in *AtPht1∶4* (*Arabidopsis* phosphate transporter 1∶4) promoter on gene expression. Experimental analyses showed that the 5′UTR intron is essential for AtPht1; 4 expression in root tips besides enhancing the level of expression in roots during Pi starvation. When 5′ UTR (112 bp) of *TaHSP26* was submitted to PlantCare database [54], we found few interesting *cis*- acting elements. One of the important elements was an imperfect CAAT box element; others are TATA box, I-box, GATA motif and CBFHV. I-box and GATA motifs are the conserved sequences present upstream of light regulated genes. They are required for light regulated tissue-specific expression. Light regulation at the transcriptional level has already been demonstrated in chloroplast targeted proteins [55]. Light has been shown to regulate the expression of small Hsps like Hsp 22 [56]. In fact, it has also been proposed that Hsp induction is primarily not because of elevated temperatures rather oxidizing environment of high light [57], and *HSP70B* also has been found to play an important role in PSII repair process [58]. Other important element found is the CBFHV which is crucial for drought stress. These CBFs are also known as dehydration-responsive-element (DRE) binding proteins (DREBs). It is a well-known fact that a high degree of overlap occurs between genes that are induced by different stresses. So, in response to one particular stress, transcription factors responsive to both the stresses have been found to be induced. DREB2A is one such transcription factor, which has been found to be one of the main regulators of drought and heat response [59]. This group found a novel splice variant of DREB2A that lacked the interacting domain (with RCD1-Radical Induced Cell Death-1) and was induced during senescence and heat shock treatment. Thus, we assume that the drastic reduction in GUS levels due to the deletion of 5′ UTR could be mainly because of the imperfect CAAT box element or the light responsive elements or the drought responsive elements present in it. It could be that one of the light/drought responsive elements has also some important role as heat stress elements as well, but is not yet characterized with respect to heat stress.

### Conclusions

Since wheat is hexaploid and the genome is large and unknown, to functionally analyse promoter of wheat chloroplast targeted sHSP26 gene for abiotic stress tolerance, *Arabidopsis* has been chosen. The results reported herein offer a picture of the mechanism underlying TaHSP26 mediated regulation of heat- tolerance *via* characterization of *TaHSP26* promoter by deletion analysis. The results provide a basis for understanding of how this *TaHSP26* promoter activity is directly related to the numbers of CCAAT box elements in promoter under heat stress. Moreover, since UTR is of primary interest in promoters, this study highlights the role of 5`UTR in enhancing GUS gene expression in heat and other abiotic stresses. In conclusion, *TaHSP26* has association with heat- tolerance in wheat and its promoter offers a possibility of inducible gene expression of otherwise abiotic stress sensitive genes in molecular breeding of superior wheat cultivars for changing environments especially high temperature.

## Materials and Methods

### Plant Material and Growth Conditions

*Arabidopsis thaliana* (Col 0) were used for raising transgenics in the present study. Wild-type seeds were spread in pots containing soilrite for the generation of full grown plants and were kept in the culture room conditions. For plating of seeds onto Murashige-Skoog (MS) medium, they were first surface sterilized with 2.0% (v/v) sodium hypochlorite for 10 minutes and then washed with sterile RO water for 3–4 times and finally suspended in 0.1% (w/v) agar. The seeds were then plated onto half-strength MS medium containing 2% sucrose and 0.8% agar, pH 5.8. The plates were kept at cold room (4°C) for 48 hr for uniform seed germination and were then kept at culture room maintained at 22±1°C with 16∶8 hr light and dark regime with a light intensity of 100–125 µmol m^−2^s^−1^_._ For raising successive generations, MS medium was supplemented with 15 mgL^−1^ hygromycin and 150 mg L^−1^ augmentin. Twenty-day mature seedlings were transferred to soil for further maturation of plants.

### Development of *TaHSP26* Promoter Deletion Constructs and Plant Transformation

The promoter region of 1625 bp was cloned in the plant transformation GATEWAY vector pMDC164 mediated by pENTR™/D-Topo cloning system [33]. Region- specific primers were designed from full length *TaHSP26* promoter and, a series of deletions were made that truncate promoter fragments thus, removing CCAAT-box elements gradually. Primers were also designed so as to remove UTR element from some of the constructs, and all the 8 constructs were PCR amplified using these region-specific primers. Sequences of the primers used are given in Table S1. All the deletion constructs were cloned into plant transformation GATEWAY ™ vector pMDC164 mediated by pENTR™/D-TOPO. The constructs were transformed in *Arabidopsis via* floral dip method [41]. At T_4_ generation, the putative transformants were confirmed for the presence of transgene of the respective deletion construct by PCR. Deletion specific primer (Forward) and GUS-reporter gene specific marker (Reverse) was used for amplification from the deletion constructs. Binary vectors harbouring various deletion fragments were used as positive control.

### High Temperature and other Abiotic Stress Treatment

Homozygous transgenic plants were germinated on half-strength MS medium in Petriplates containing 15 mg L^−1^ hygromcyin. Two-week-old full seedlings of 8 different deletion constructs were subjected to heat stress at 37°C for 2 hrs and then analysed histochemically. From soil grown mature plants, various tissues (leaf, root, stem, flower, young siliques, mature siliques) were harvested after high temperature treatment at 37°C for 2 hrs. Five individual transgenic plants from each line were analyzed. For time-course experiment, three-week old transgenic seedlings were subjected to heat stress of 37°C for different time-points. For each experiment after heat-stress, the tissue was analyzed by GUS histochemical assay and the tissue was also frozen in liquid nitrogen and stored at −80°C for RNA and protein isolation. For drought stress, salt stress and cold stress, two-week old seedlings were subjected to 2% mannitol for 24 hrs, 150 mM NaCl for 24 hrs and 4°C for 24 hrs respectively and then analyzed.

### Histochemical GUS Assay

Histochemical GUS staining was performed as described in the protocol by Jefferson et al. [60]. The tissues were first given heat stress at 37°C for 2 hrs. and then analyzed for GUS staining. The tissues used were seedling tissue, leaf, root, stem, flower, young silique, mature silique. Transgenic plant without heat stress was used as control. All the samples were incubated at 37°C for 24 hrs in the GUS buffer. After the GUS staining, tissues were treated with ethanol and acetic acid (3∶1) to remove chlorophyll from the GUS stained tissue.

### Quantitative RT-PCR Analysis

Total RNA was isolated from different tissues of two-week old *Arabidopsis* transgenic seedlings and other tissues from mature plants i.e. Leaf, root, stem, flower, young siliques, mature siliques using RNeasy plant mini kit (Qiagen, Germany) according to the manufacturer’s instructions including on-column DNase-I treatment to remove genomic DNA contamination. GUS transcript was analysed in all the deletion constructs. First strand cDNA was synthesized from 2 µg of total RNA employing the high-capacity cDNA archive kit (Applied Biosystems, USA). Reaction constitutes cDNA samples along with 200 nM of each primer and SYBR Green PCR master mix (Applied Biosystems, USA) and run on the ABI Prism 7000 Sequence Detection System and Software (PE Applied Biosystems). Amplification of cDNA was confirmed by melting curve analysis. *Actin*, was used as an internal control for the quantification of mRNA levels of the constructs. All reactions had at least three biological and three technical replicates.

### Fluorimetric GUS Assay

The transgenic lines which were positive for GUS histochemical staining were taken for further fluorimetric analysis. Total protein was extracted from the T_4_ transgenic plants and final concentration of 6 µgm protein was taken for estimation of GUS protein. Fluorimetric assay was done according to Jefferson et al. [60]. The substrate used was 4-methylumbelliferyl β-D-glucuronide. The protein was incubated with the substrate at 37°C for 15 hrs. and the reaction was stopped by 0.2 M Na_2_CO_3_ in the dark. The reaction product 4-methyl umbelliferone (4-MU) was estimated by a DyNA Quant TM 200 fluorimeter (Hoefer Pharmacia Biotech Inc., California, USA). The assay was done in triplicates for each of the biological sample taken.

### Statistical Analyses

Statistical analysis was done by calculating mean value for all the three replicates. Standard error was also calculated based on these replicates. Readings were calculated in pmol/2 ml. The final readings were calibrated in nmol/mg/hr.

## Supporting Information

Figure S1 **The sequence of the putative promoter region of HSP26.** Some *cis*- acting elements have been highlighted in the putative promoter sequence through PLACE promoter motif analysis. Three CCAAT BOX1 elements (721, 1209, 1435) are highlighted in green, two of them lie in the antisense-strand while one of them lie in the sense strand.
(TIF)

Figure S2 **PCR amplification of different deletions of wheat** ***TaHSP*** **26 promoter for TOPO cloning.**
(TIF)

Figure S3 **Analysis of control and heat-stressed (a) leaf tissue, and (b) root tissue of** ***Arabidopsis*** **transgenic plants harboring full** ***TaHSP26*** **promoter under bright field using fluorescence microscope for histochemical localization of GUS activity in control and heat stress tissues, respectively.**
(TIF)

Table S1 **List of primers used.**
(DOCX)
