# Supplementary figures and images for: Wheat Chloroplast Targeted sHSP26 Promoter Confers Heat and Abiotic Stress Inducible Expression in Transgenic Arabidopsis Plants
Source: PLoS One. 2013 Jan 18;8(1):e54418. doi: 10.1371/journal.pone.0054418 (PMC3548792; doi:10.1371/journal.pone.0054418)

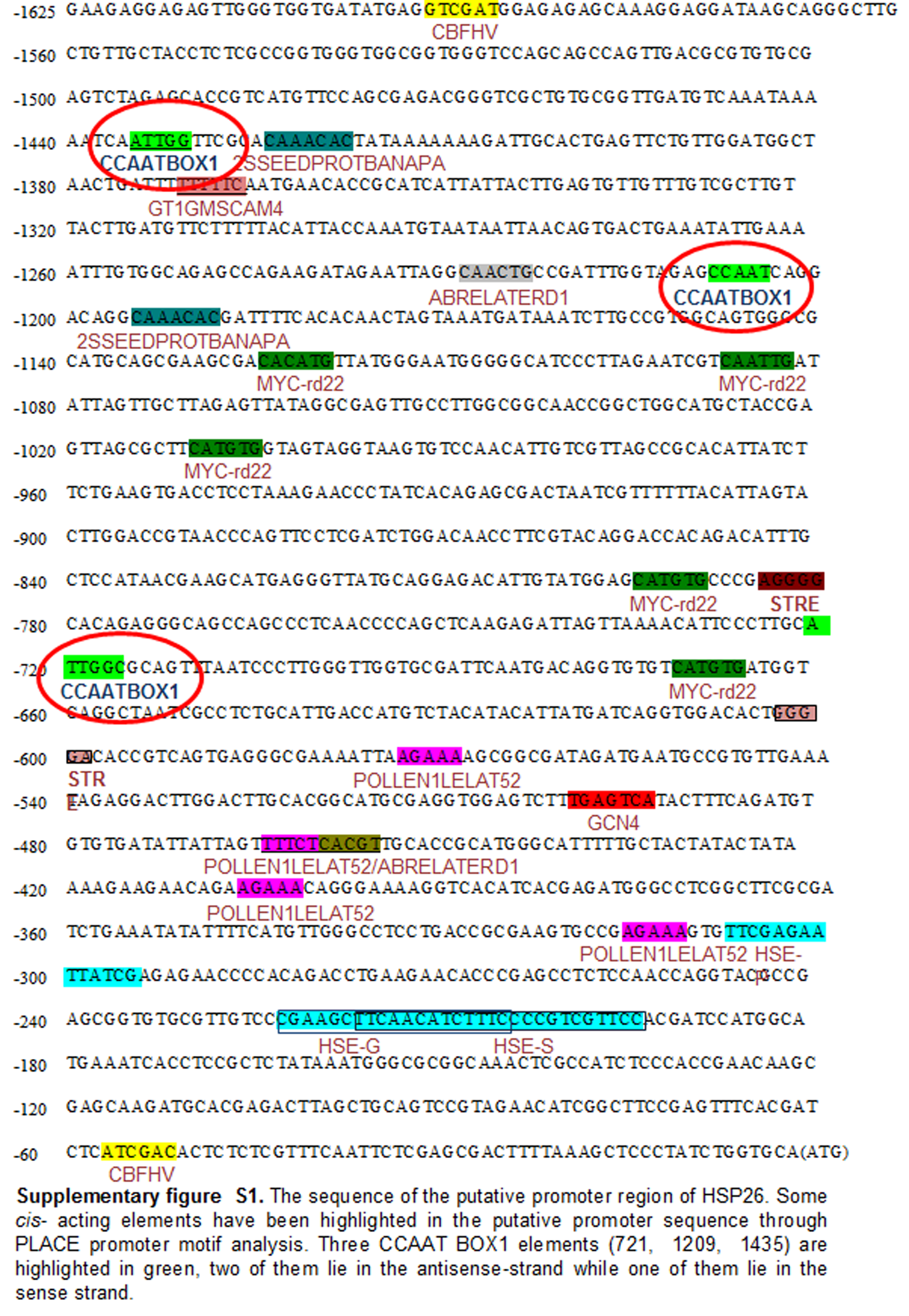

Supplement: Figure S1 — The sequence of the putative promoter region of HSP26. Some cis- acting elements have been highlighted in the putative promoter sequence through PLACE promoter motif analysis. Three CCAAT BOX1 elements (721, 1209, 1435) are highlighted in green, two of them lie in the antisense-strand while one of them lie in the sense strand. (TIF) [file pone.0054418.s001.tif]

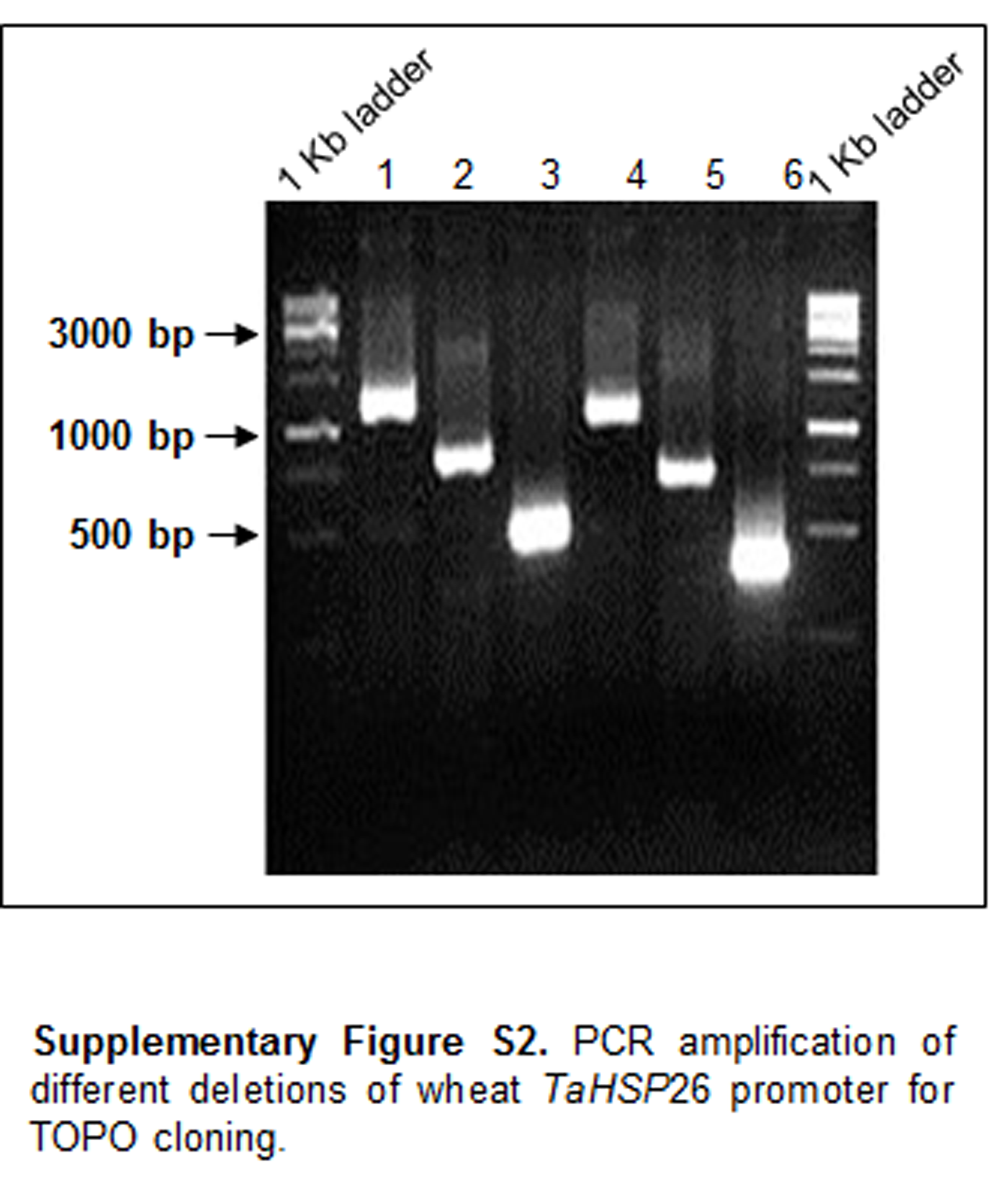

Supplement: Figure S2 — PCR amplification of different deletions of wheat TaHSP 26 promoter for TOPO cloning. (TIF) [file pone.0054418.s002.tif]

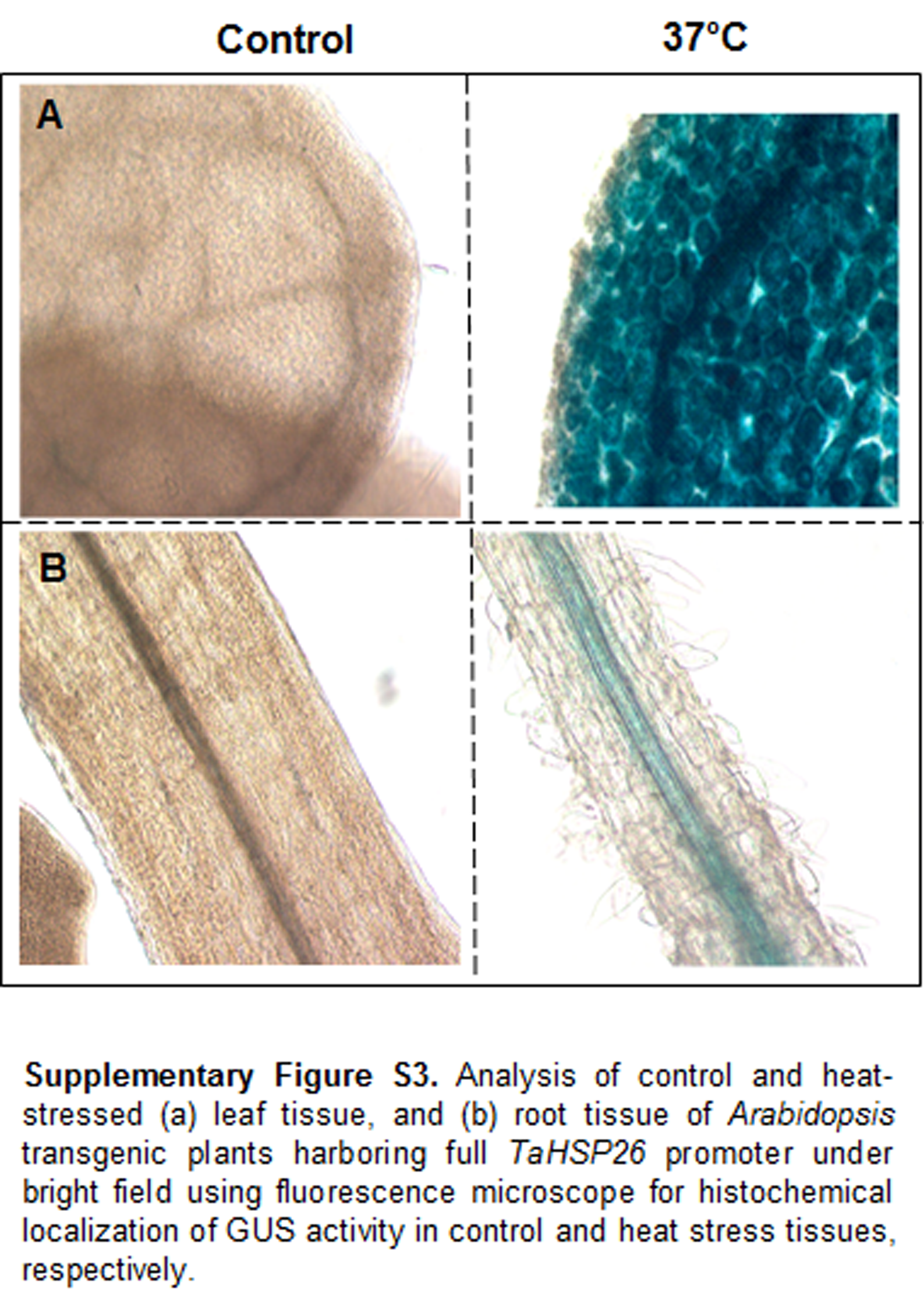

Supplement: Figure S3 — Analysis of control and heat-stressed (a) leaf tissue, and (b) root tissue of Arabidopsis transgenic plants harboring full TaHSP26 promoter under bright field using fluorescence microscope for histochemical localization of GUS activity in control and heat stress tissues, respectively. (TIF) [file pone.0054418.s003.tif]
