# Supplementary material for: Wheat Chloroplast Targeted sHSP26 Promoter Confers Heat and Abiotic Stress Inducible Expression in Transgenic Arabidopsis Plants
Source: PLoS One. 2013 Jan 18;8(1):e54418. doi: 10.1371/journal.pone.0054418 (PMC3548792; doi:10.1371/journal.pone.0054418)
Supplement: Table S1 — List of primers used. (DOCX) [file pone.0054418.s004.docx]

**Table S1**: List of primers used

| **Primer ID** | **Primer Sequence (5’-3’)** |
| --- | --- |
| At Actin Real time F | TGTGCTCAGTGGTGGAACCA |
| At Actin Real time R | GAGCCAAAGCAGTGATCTCTTTG |
| P26+UTR | TCCCCCGGGTGCACCAGATAGGGAGC |
| P26-UTR | TCCCCCGGGATCTTGCTCGCTTGTTCGG |
| P26_Del 1 | CACCCCAAATGTAATAATTAACAGTG |
| P26_Del 2 | CACCAGTTCCTCGATCTGGACAAC |
| P26_Del 3 | CACCGGACTTGCACGGCATGC |
